# Global research trends and collaborations in acute kidney injury (AKI) and sepsis: a bibliometric analysis (2004–2024)

**DOI:** 10.1080/0886022X.2025.2494049

**Published:** 2025-04-24

**Authors:** Yuru Yang, Shuang Zhao, Shuai Liu

**Affiliations:** a Department of Nephrology, Shibei Hospital of Jing’an District, Shanghai, PR China; b Department of Nephrology, Shanghai Municipal Hospital of Traditional Chinese Medicine, Shanghai University of Traditional Chinese Medicine, Shanghai, PR China

**Keywords:** Acute kidney injury (AKI), acute kidney failure (ARF), sepsis, bibliometric analysis

## Abstract

**Background:**

Acute kidney injury (AKI) and sepsis are critical clinical conditions associated with high morbidity and mortality. Despite growing research interest, there remains a need for a comprehensive analysis of global research trends in this field. Bibliometric analysis offers a quantitative approach to assessing the evolution of scientific knowledge, collaborative networks, and emerging research areas over time.

**Objective:**

This study aims to map the global landscape of research on AKI and sepsis over the last two decades (2004–2024), identify major contributors, collaboration networks, key research trends, and highlight gaps in the literature.

**Methods:**

We conducted a bibliometric analysis of research articles from leading databases. The study utilized network visualization techniques to assess co-authorship, citation patterns, and keyword co-occurrence, focusing on the most influential countries, institutions, and research collaborations.

**Results:**

Results reveal China leads in publication volume, yet countries like the United States and Australia show higher international collaboration rates and citation impact. Additionally, thematic analyses highlight critical research areas, including biomarkers, bioenergetics, inflammation, and machine learning, marking significant advancements in the understanding and management of AKI.

**Conclusion:**

This bibliometric analysis offers valuable insights into the evolving landscape of AKI and sepsis research, emphasizing the importance of collaborative efforts to address knowledge gaps and ensure evidence-based care across diverse healthcare settings. Future research should prioritize the development of biomarkers and the integration of AI-driven technologies to enhance early diagnosis and personalize treatment strategies for AKI patients.

## Introduction

Acute kidney injury (AKI) is a critical condition frequently encountered in critically ill patients, with sepsis being one of its most common and severe causes, accounting for up to 50% of cases in the intensive care unit (ICU) setting [1–3]. AKI associated with sepsis significantly increases patient mortality and morbidity, posing a considerable challenge for clinicians due to its rapid onset and severe complications. When sepsis-induced AKI occurs, it often leads to multi-organ dysfunction, prolonged ICU stays, and a higher risk of long-term kidney impairment [4]. The pathophysiology of sepsis-induced AKI is highly complex, involving multiple interrelated mechanisms, including systemic inflammation, oxidative stress, microcirculatory dysfunction, endothelial injury, and tubular cell damage [5]. These pathological processes disrupt kidney perfusion, alter cellular metabolism, impair oxygen delivery, and ultimately lead to cellular injury and apoptosis [5].

Over the past two decades, there has been a notable surge in research focused on AKI, particularly in the context of sepsis, driven by the increasing prevalence of this condition in critical care settings [6]. Pioneering studies have provided new insights into how sepsis-induced AKI develops, exploring the role of immune responses, hemodynamic alterations, and cellular bioenergetics in the onset and progression of kidney injury [7]. For instance, research has revealed that systemic inflammation in sepsis triggers a cascade of events that damage the kidney’s endothelial cells and disrupt the microcirculatory network, leading to impaired oxygen delivery and metabolic dysregulation [8]. Moreover, the identification of key biomarkers, such as neutrophil gelatinase-associated lipocalin (NGAL) and procalcitonin, has advanced the early diagnosis of AKI, aiding in timely intervention and better patient outcomes [9,10].

Given the substantial body of literature now available on AKI and sepsis, it is important to systematically assess and map the development of knowledge in this field. Bibliometric analysis is a valuable tool for this purpose, as it provides valuable insights into the evolution of research trends, influential contributors, and collaboration networks within a given field [11–13]. The purpose of performing a bibliometric analysis in the context of AKI and sepsis is to provide a comprehensive overview of the research landscape. It helps quantify the contribution of different countries, institutions, and researchers to the field, thereby identifying global leaders in AKI and sepsis research. By understanding which regions and institutions are at the forefront of this research, it becomes possible to foster more targeted collaborations and to drive further innovation in the field. Additionally, this analysis can reveal emerging research topics that are gaining traction, helping to guide future research efforts in the direction of the most pressing clinical questions.

## Materials and methods

### Data collection

**Figure 1.**
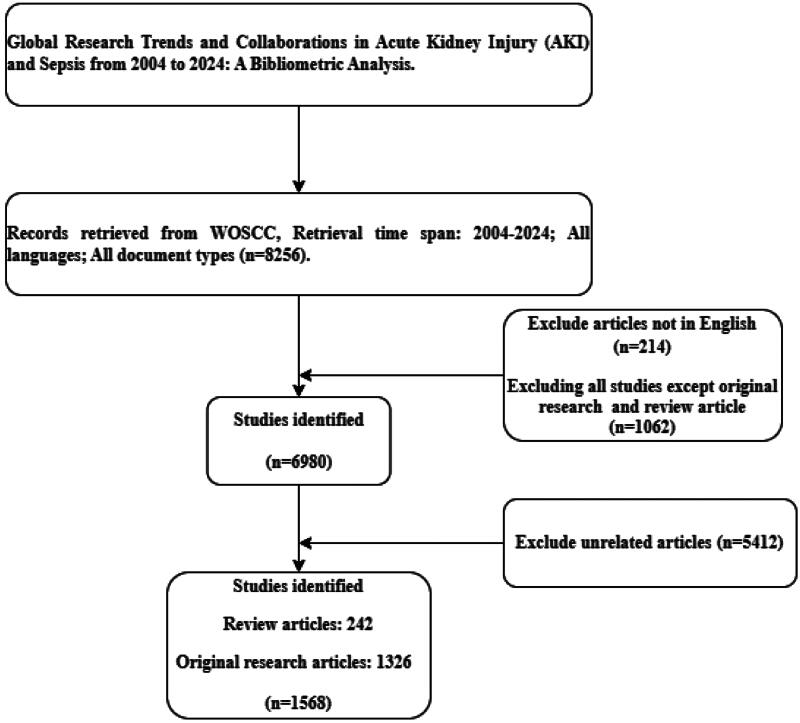
Detailed literature screening process.

We systematically searched the Web of Science Core Collection (WoSCC) on September 5, 2024, using the following keywords: (‘acute renal failure’ OR ‘acute renal injury’ OR ‘acute kidney injury’ OR ‘acute kidney failure’ OR ‘AKI’ OR ‘ARF’) AND ‘sepsis’. This search strategy was designed to capture all relevant studies across clinical trials, reviews, and experimental research related to AKI and sepsis. The search spanned publications from January 1, 2004, to September 5, 2024. Two authors conducted independent searches, reviewed titles, and, when necessary, read abstracts. Disagreements were resolved through discussion, achieving a 90% agreement rate. Non-English articles were excluded, and the search was limited to reviews and research articles, yielding 1,568 publications in XML format from WoSCC for further analysis. Of these, 242 (15.4%) are review articles and 1,326 (84.6%) are original research articles. The research process is detailed in Figure 1. This strategy was chosen to capture studies across all relevant categories, including clinical trials, reviews, and experimental research. WoSCC was selected due to its comprehensive citation tracking capabilities, providing a clear picture of the global research landscape. However, this search strategy is limited by the exclusion of other significant databases such as Scopus and PubMed, which may contain relevant articles not indexed in WoSCC. Furthermore, our analysis only included English-language publications, potentially introducing bias by excluding studies published in other languages.

### Data analysis and visualization

In this study, data were retrieved from the WoSCC, including key indicators such as publication counts, citation metrics, H-index, G-index, authors, institutions, countries, journals, references, and keywords. The dataset was exported in TXT format for processing. To assess research performance, we employed R 4.3.1 (R Foundation for Statistical Computing, Vienna, Austria) [14] with the bibliometrix package, computing citation metrics, H-index, and G-index to evaluate the productivity and impact of various research entities. The annual publication trend was analyzed to capture the field’s growth, while citation patterns were examined to track the influence of key studies over time. Journal impact was assessed using Impact Factor (IF) and Journal Citation Reports (JCR) extracted from Web of Science.

To analyze research collaboration, we used co-authorship network analysis, visualized through the igraph package in R and VOSviewer (Centre for Science and Technology Studies, Leiden University, Netherlands) [15]. Nodes in these networks represent authors, institutions, or countries, while edges indicate collaborative strength. Institutional collaborations were mapped using VOSviewer, where node sizes reflect publication volume, and linkages highlight research partnerships. Different colors represent different clusters. Additionally, keywords were grouped based on their co-occurrence using VOSviewer, revealing dominant and emerging themes within the sepsis-induced AKI research landscape.

To further investigate the evolution of research themes, we conducted temporal and trend analysis using CiteSpace 6.2.R4 (Chaomei Chen, Drexel University, USA) [16] Research trends were assessed through time slicing (1-year intervals), while citation burst detection (threshold: 5 publications) identified influential studies with sudden increases in citations. This approach helped track thematic shifts and highlight emerging research directions.

## Results

### Global publication trend

**Figure 2.**
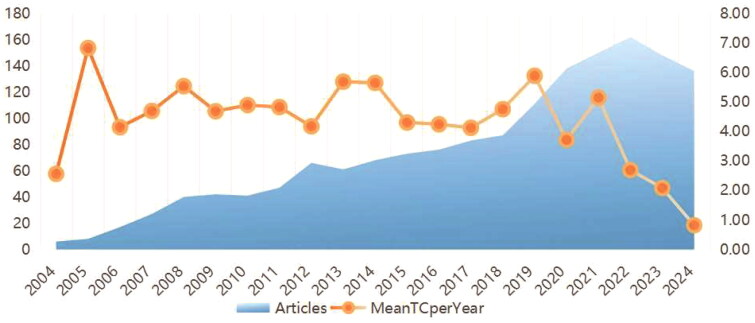
Distribution of citations and publications in the field of sepsis-induced AKI from 2004 to 2024.

A total of 1,587 publications were identified from 2004 to 2024. The annual number of published articles (represented by a blue area) and the mean total citations per year (MeanTCperYear, shown as orange circles) from 2004 to 2024 are illustrated in Figure 2. The number of publications grew steadily from 2004 to 2008, plateaued from 2008 to 2010, and then resumed growth, peaking between 2020 and 2022. Additionally, the citation peak occurred around 2005 (Figure 2).

### Contribution of countries

Eighty-four countries have engaged in research on AKI and sepsis. The results of Table 1 reveal notable patterns in global research output and collaboration in the studied field. China leads with 562 articles, representing 35.4% of the total, but shows a relatively low rate of international collaboration, with only 8% of its publications being multi-country publications (MCP). In contrast, the United States, while contributing 282 articles (17.8%), has a significantly higher MCP percentage (23.4%), indicating more frequent international cooperation. Japan, with 63 articles, follows a similar pattern to China, contributing 11.1% MCP. Australia stands out for its strong international partnerships, with 61% of its 41 publications being MCPs. This suggests a high degree of collaboration with foreign institutions. Similarly, Italy (27.7%), Germany (30%), and the Netherlands (35.3%) show a strong emphasis on multi-country collaborations, reflecting their active roles in global research networks. On the other hand, India, with 46 articles, exhibits a strong domestic research focus, with only 2.2% MCP, indicating limited international collaboration. Although China published the most articles, its average citation rate was relatively low (17.5), while countries like Canada (102.6), Australia (82.9), the U.S. (65.9), and France (51.6) had significantly higher citation rates (Table 1).

**Table 1.** The top ten published country.

Country	Articles	% Of total	SCP	MCP	MCP ratio	Total citations	Average article citations
China	562	35.4	517	45	8	9818	17.5
USA	282	17.8	216	66	23.4	18584	65.9
Japan	63	4	56	7	11.1	1318	20.9
Australia	54	3.4	52	2	3.7	3397	82.9
Italy	48	3	42	6	12.5	1687	35.9
France	47	3	34	13	27.7	1495	51.6
Germany	46	2.9	45	1	2.2	1679	42
South Korea	41	2.6	16	25	61	956	17.7
Canada	40	2.5	28	12	30	3180	102.6
Netherlands	34	2.1	22	12	35.3	1532	45.1

SCP: single country publications, MCP: multiple country publications.

**Figure 3.**
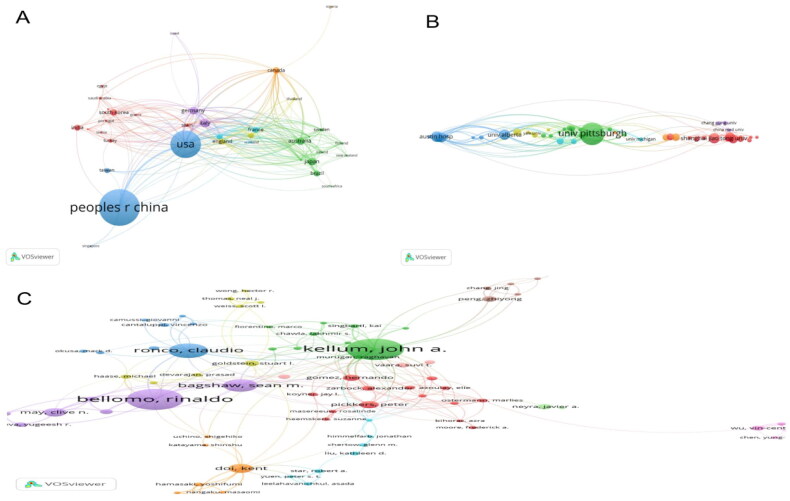
(A) Network of co-authorship between countries. (B) Network of co-authorship between institutions. (C) Network of co-authorship between authors. Node size corresponds to the number of published articles, while different colors denote distinct clusters.

We established a co-authorship network among countries and set a minimum threshold of 5 published articles for inclusion in the VOSviewer analysis. Out of 84 countries, 42 met this criterion, with the largest connected network comprising 38 items. Node size corresponds to the number of published articles, where larger nodes indicate higher publication counts. The connecting lines represent collaborative relationships between countries, while different colors denote distinct clusters. The USA appeared as the central node, indicating its substantial role in AKI and sepsis research and extensive collaborations with countries like Germany, China, Italy, and Canada. China, despite its high output, showed less dense collaborations, particularly with European countries. Strong collaboration networks were observed among European nations such as the UK, France, Germany, and Italy (Figure 3A).

### Contribution of institutions

The top 10 institutions are listed in Table 2. The results show the leading institutions contributing to research output. The Pennsylvania Commonwealth System Of Higher Education (PCSHE) ranks first with 157 articles, accounting for 9.89% of the total. Following closely is the University of Pittsburgh, which has produced 146 articles, making up 9.2% of the total output. The Florey Institute of Neuroscience and Mental Health in Australia ranks third with 85 articles (5.36%). In fourth place, the University of California System contributed 63 articles (3.97%), while Capital Medical University in China follows with 57 articles (3.59%). Other notable institutions include the Austin Research Institute and the Egyptian Knowledge Bank (EKB), both producing 55 articles (3.47%). Central South University in China and Universite Paris Cite in France have each published 51 articles, comprising 3.21% of the total output. Finally, National Taiwan University in China produced 50 articles, accounting for 3.15% of the research in this area. Of the top institutions, four were from the USA, three from China, and one each from Australia, France, and Egypt. These results highlight the dominance of institutions from the USA, China, and Australia in contributing to the field, with a few key contributions from France and Egypt (Table 2).

**Table 2.** The top 10 institutions with the highest number of publications.

Rank	Institution	Country	Articles	% Of total
1	Pennsylvania Commonwealth System Of Higher Education (PCSHE)	USA	157	9.89
2	University Of Pittsburgh	USA	146	9.20
3	Florey Institute of Neuroscience and Mental Health	Australia	85	5.36
4	University of California System	USA	63	3.97
5	Capital Medical University	China	57	3.59
6	Austin Research Institute	USA	55	3.47
7	Egyptian Knowledge Bank (EKB)	Egypt	55	3.47
8	Central South University	China	51	3.21
9	Universite Paris Cite	France	51	3.21
10	National Taiwan University	China	50	3.15

The institutional collaboration network, visualized using VOSviewer, illustrates the intricate connections among key research institutions engaged in sepsis-induced AKI research. The analysis included institutions that had published at least 25 articles, identifying 55 institutions that collaborated on more than ten publications. After filtering out unconnected institutions, 51 instances of institutional collaboration were observed. Among the institutions, the University of Pittsburgh emerges as the most influential, boasting the highest number of collaborative links, particularly with the University of Michigan, the University of Cincinnati, and St. Bortolo Hospital. This central positioning underscores its pivotal role in fostering international research collaborations. Institutions from North America and Europe, such as the University of Alberta, the University of Colorado, Austin Hospital and Helsinki University Hospital, form a well-connected cluster, reflecting robust collaboration among high-income countries. In contrast, Asian institutions, including Wuhan University, Shanghai Jiao Tong University, Capital Medical University and Chang Gung University, demonstrate a distinct collaboration pattern, primarily engaging with regional partners (Figure 3B).

### Analysis of authors

The results of Table 3 show that John A. Kellum ranks first in terms of productivity and impact, with 54 published papers (NP), a total of 1,552 citations (TC), an H-index of 35, and a G-index of 54. This highlights his influential role in the field of AKI research. Rinaldo Bellomo follows closely with 52 papers, 1,283 citations, and an H-index of 33, showing his significant contributions alongside Kellum. Claudio Ronco and Sean M. Bagshaw also rank highly, with 35 and 30 papers, respectively, and demonstrate strong citation impact with H-indices of 20 and 24. In contrast, Zhang L, Zhang Y, and Zhang J have lower citation counts, indicating emerging contributions, but still maintain consistent output with 20-23 papers each. This suggests a diverse range of productivity levels, with Kellum, Bellomo, and Ronco leading the field, while others are developing their influence (Table 3). We analyzed the authors’ partnership network using VOSviewer software. Authors included in the analysis were those who had published at least 5 articles, identifying 122 authors who collaborated on more than five publications. After excluding authors who were not connected, 77 instances of author collaboration were found. The three leading authors with the highest overall connections were Bellomo R (total link = 97), Kellum JA (total link = 95), Bagshaw SM (total link = 64) (Figure 3C). Figure 3(C) shows Kellum JA emerges as a central figure, from the University of Pittsburgh School of Medicine (USA), collaborates closely with Murugan R from the same institution, highlighting strong intra-institutional ties. He also works with Vaara ST of the University of Helsinki (Finland), reflecting international partnerships driven by common research interests in critical care nephrology. Similarly, Bellomo R, based at the University of Melbourne (Australia), shows extensive co-authorship links with Bagshaw SM from the University of Alberta (Canada) and Ronco C from San Bortolo Hospital in Italy. These collaborations likely result from a mix of shared institutional backgrounds, regional research networks, and international initiatives focused on advancing the understanding and treatment of sepsis-induced acute kidney injury (Supplementary table 1).

**Table 3.** Top 10 most published authors on the field of sepsis-induced AKI.

Rank	Authors	NP	NC	H-index	G-index
1	Kellum JA	54	1552	35	54
2	Bellomo R	52	1283	33	52
3	Ronco C	35	654	20	35
4	Bagshaw SM	30	1174	24	30
5	Zhang L	23	76	11	23
6	Zhang Y	23	55	9	16
7	Doi K	20	61	14	20
8	Zhang J	20	59	9	17
9	May CN	18	300	18	18
10	Li Y	16	22	7	15

NP: no. of publishes, NC: no. of citations.

### Analysis of journals

The top 10 journals, shown in Table 4. The results highlight that *Critical Care* is the most prominent journal in the field, with 55 publications and an impressive 3,635 citations. It boasts the highest H-index of 35, reflecting its significant impact in the domain, further supported by an IF of 8.8 in 2023. *Renal Failure* and *BMC Nephrology* follow, with 51 and 34 publications, respectively. However, their citation counts and H-indices are notably lower, indicating a smaller yet relevant impact in nephrology. Journals such as *American Journal of Physiology-Renal Physiology* and *Shock* have fewer publications (31 and 30, respectively), but their higher citation counts (1318 and 975) and H-indices (22 and 15) suggest that the papers published there are highly regarded. *Kidney International* and *Intensive Care Medicine* also demonstrate strong influence, with IFs of 14.8 and 29.6, respectively, making them key journals in this field. Overall, *Critical Care* and *Kidney International* emerge as leading journals for high-impact research in AKI and critical care nephrology, while others like *Renal Failure* and *Shock* contribute valuable, more specialized insights (Table 4).

**Table 4.** Summary of the top 10 journals in terms of publications.

Rank	Journal	NP	NC	NO. of total	H-index	G-index	IF (2023)	JCR (2023)
1	Critical Care	55	3,635	3.47	35	55	8.8	1.97
2	Renal Failure	51	500	3.21	17	27	3.1	1.02
3	BMC Nephrology	34	401	2.14	13	22	2.2	0.83
4	Journal Of Critical Care	34	448	2.14	19	29	3.2	0.73
5	American Journal Of Physiology-Renal Physiology	31	1,318	1.95	22	31	3.7	1.32
6	Shock	30	975	1.89	15	30	2.7	0.96
7	Critical Care Medicine	28	3,619	1.76	23	28	7.7	1.75
8	Kidney International	28	3,566	1.76	25	28	14.8	4.79
9	Plos One	27	930	1.70	17	27	2.9	0.88
10	Nephrology Dialysis Transplantation	26	1,279	1.64	19	26	4.8	1.84

NP: no. of publishes, NC: no. of citations.

### Analysis of keywords

**Figure 4.**
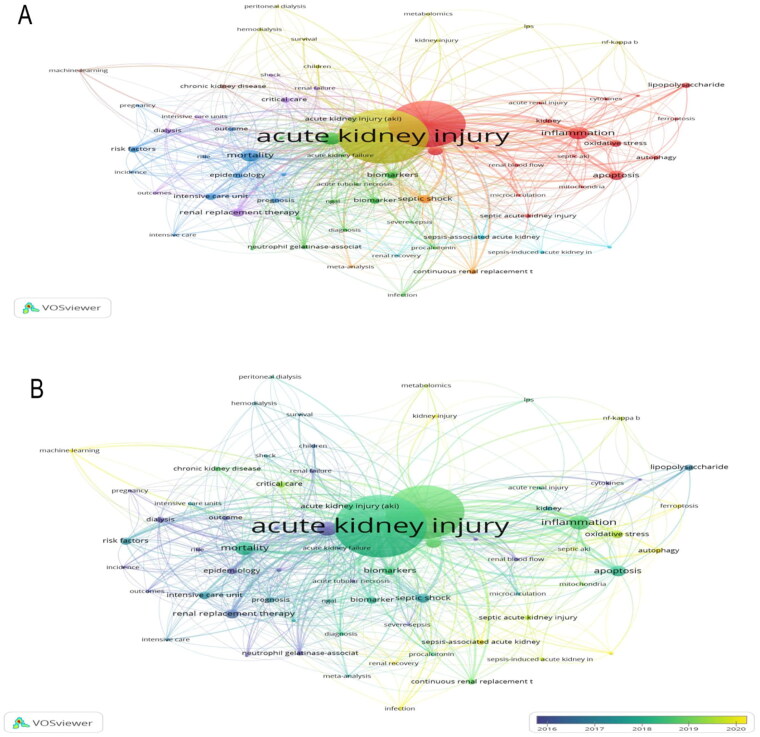
Keyword co-occurrence analysis. (A) Keyword co-occurrence network, colors represent different research clusters, such as biological mechanisms (red), biomarkers (green), clinical outcomes (blue), and critical care interventions (purple/yellow). (B) Keyword trends from 2016 to 2020, with the color gradient at the bottom representing the years.

Table 5 displays the most frequent keywords, led by ‘acute renal failure’, ‘critically-ill patients’, and ‘sepsis’. To analyze keywords more clearly, we conducted a keyword analysis with VOSviewer software (Figure 4). The number of keyword co-occurrences was set to be no less than 10, identifying 69 core keywords categorized into eight clusters. Cluster 1 (Red)—Mechanisms of AKI: This cluster focuses on the biological mechanisms involved in AKI, particularly in the context of sepsis-induced AKI. It highlights key processes such as inflammation, oxidative stress, and apoptosis, which play critical roles in the development of AKI. Cluster 2 (Green)—Diagnosis and Biomarkers for AKI: This cluster emphasizes clinical biomarkers for AKI diagnosis, including creatinine and NGAL. These biomarkers are crucial for early detection and accurate diagnosis of AKI, particularly in critically ill patients. Cluster 3 (Blue)—Epidemiology and Outcomes of AKI: This cluster addresses the epidemiology and outcomes of AKI, focusing on mortality, incidence, and risk factors. It provides insights into the global burden of AKI and its impact on patient survival, emphasizing the importance of early identification and intervention. Cluster 4 (Yellow)—Pediatric AKI and Dialysis: This cluster highlights pediatric AKI, particularly focusing on peritoneal dialysis and metabolomics. It sheds light on the unique challenges of managing AKI in children and the role of advanced diagnostic techniques in understanding pediatric kidney injury. Cluster 5 (Purple)—Critical Care and Renal Replacement Therapy (RRT): This cluster explores critical care interventions for AKI, particularly the use of dialysis and RRT. It underscores the importance of timely interventions in critically ill patients to improve outcomes in cases of severe AKI. Cluster 6—Cell Death Mechanisms and Sepsis-Induced AKI: This cluster emphasizes the mechanisms involved in sepsis-associated and sepsis-induced AKI, particularly focusing on apoptosis and renal recovery. It highlights how inflammatory cell death contributes to kidney injury and recovery processes during sepsis. Cluster 7—Renal Replacement Therapy (CRRT) and Meta-Analysis: This cluster revolves around the use of continuous renal replacement therapy CRRT in the management of AKI and septic shock. It also includes meta-analyses that evaluate the effectiveness of various interventions for improving AKI outcomes in critical care settings. Cluster 8—Chronic Kidney Disease (CKD) and Machine Learning: This cluster explores broader themes related to CKD and the integration of machine learning in clinical applications. It focuses on how artificial intelligence AI can enhance personalized care for AKI patients, aiding in early detection, risk stratification, and treatment decisions (Figure 4A). The network visualization of Figure 4(B) highlights the key terms and relationships related to ‘AKI’ research from 2016 to 2020. The central and most prominent node is ‘acute kidney injury,’ indicating its dominance as a focal point in the literature. Key concepts such as ‘biomarkers,’ ‘inflammation,’ and ‘apoptosis’ are shown in green, signifying more recent research trends (around 2020), whereas older terms like ‘mortality’ and ‘epidemiology’ in blue indicate established areas of study (2016) (Figure 4B).

**Table 5.** Ten most frequently occurring keywords in the study.

Rank	Words	Occurrences
1	Acute-renal-failure	693
2	Critically-ill patients	427
3	Sepsis	347
4	Mortality	322
5	Septic shock	221
6	Acute kidney injury	172
7	Disease	154
8	Outcomes	152
9	Epidemiology	148
10	Failure	135

**Figure 5.**
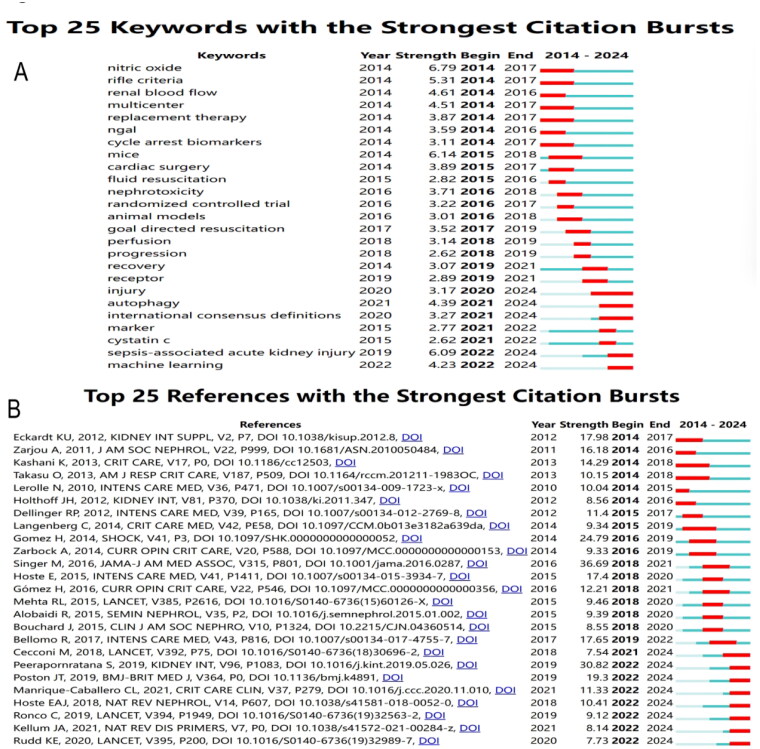
(A) Top 25 keywords for the strongest citation bursts. (B) Top 25 references for the strongest citation bursts.

The chart of Figure 5(A) presents the Top 25 Keywords with the strongest citation bursts from 2014 to 2024, showing the most rapidly emerging research themes in acute AKI and related areas. The keywords nitric oxide, RIFLE criteria, and renal blood flow saw strong citation bursts between 2014 and 2017, reflecting an initial focus on key physiological mechanisms and clinical guidelines for AKI management. In more recent years, terms like machine learning (2022–2024) and sepsis-associated acute kidney injury (2019–2024) indicate a growing interest in leveraging AI technologies and understanding the links between sepsis and AKI. Similarly, autophagy (2021–2024) and receptor (2019–2021) highlight attention on cellular and molecular mechanisms underlying AKI (Figure 5A).

### Top six most cited references

The top six most cited references in Table 6 cover critical research in AKI, sepsis, and associated outcomes in critically ill patients. These influential papers shape current understanding of AKI pathophysiology, diagnostics, and treatment, particularly in septic patients. Acute renal failure in critically ill patients—A multinational, multicenter study: This landmark study provides detailed data on the prevalence, outcomes, and management strategies for AKI in critically ill patients, highlighting the global burden of AKI and the need for improved interventions [2]. By showing the global burden of AKI, this study reinforced the need for standardized management protocols and better diagnostic and therapeutic interventions. The Third International Consensus Definitions for Sepsis and Septic Shock: This widely-cited consensus paper redefines sepsis and septic shock, establishing new criteria for diagnosis, which is critical for clinicians managing septic patients with AKI [1]. The paper redefined the criteria for sepsis (Sepsis-3), influencing clinical diagnostics. Acute renal failure - definition, outcome measures, animal models, fluid therapy, and information technology needs: The Second International Consensus Conference of the ADQI Group: This consensus report lays out foundational concepts in AKI research, offering guidance on definitions, animal models, and future research directions for improving AKI treatment [17]. The paper established foundational definitions and guidelines for AKI management, driving clinical standardization. Mechanisms of disease: Acute renal failure and sepsis: This paper explores the mechanistic links between sepsis and AKI, focusing on inflammation, hemodynamic instability, and microcirculatory dysfunction as critical drivers of renal failure in septic patients [18]. The paper helped clarify the pathophysiological mechanisms of sepsis-induced renal failure, offering critical insights into potential therapeutic targets. Septic acute kidney injury in critically ill patients: Clinical characteristics and outcomes: This paper provides a detailed clinical analysis of septic AKI, highlighting its unique characteristics, the challenges in management, and its association with higher mortality rates [19]. This paper helped to define the clinical presentation and outcomes of septic AKI, emphasizing the need for early recognition and intervention. Acute Kidney Injury Network: Report of an initiative to improve outcomes in acute kidney injury: This report from the Acute Kidney Injury Network (AKIN) focuses on improving the standardization of AKI definitions and treatment protocols to enhance outcomes for patients globally [20]. This initiative was instrumental in standardizing the diagnosis and treatment of AKI worldwide.

**Table 6.** Top 6 most cited references on field of sepsis-induced AKI.

Rank	Titles	Authors	Journals	Publish year	Total citations	Total link strength
1	Acute renal failure in critically ill patients - A multinational, multicenter study	Uchino S	JAMA-J AM MED ASSOC	2005	442	5,400
2	The Third International Consensus Definitions for Sepsis and Septic Shock	Singer M	JAMA-J AM MED ASSOC	2016	316	3,033
3	Acute renal failure - definition, outcome measures, animal models, fluid therapy and information technology needs: the Second International Consensus Conference of the Acute Dialysis Quality Initiative (ADQI) Group	Bellomo R	CRIT CARE	2004	296	3,746
4	Mechanisms of disease: Acute renal failure and sepsis	Schrier RW	NEW ENGL J MED	2004	230	2,485
5	Septic acute kidney injury in critically ill patients: Clinical characteristics and outcomes	Bagshaw SM	CLIN J AM SOC NEPHRO	2007	227	3,334
6	Acute Kidney Injury Network: report of an initiative to improve outcomes in acute	Mehta RL	CRIT CARE	2007	212	2,577

The Figure 5(B) presents the top 25 references in the field with the strongest citation bursts between 2014 and 2024, indicating the most influential works during this period. The highest citation burst strength is attributed to the study by Singer M (2016) published in *JAMA* [1], reflecting significant influence from 2018 to 2021. Another prominent work is by Gomez H (2014) in *Shock* [21], with a burst from 2016 to 2019, indicating sustained relevance in critical care and shock-related research. More recent articles, such as those by Peerapornratana S (2019) [5] and Poston JT (2019) [4], show bursts extending into 2024, highlighting ongoing relevance in acute kidney injury and nephrology. This trend suggests a shift toward recent publications maintaining strong impacts in nephrology and critical care, indicating an evolving focus in the literature over time (Figure 5B).

## Discussion

### Global trends and research output

The annual publication trends indicate a steady increase in the number of studies. The mean total citations per year peaked around 2005, indicating that studies published during this period have maintained a lasting academic influence. Furthermore, citations tend to accumulate over time, with earlier publications having a longer window to garner citations, which may explain the higher mean total citations per year observed in the earlier years. Our analysis of country-wise contributions reveals that high-income countries, particularly the USA, Australia, and European nations, dominate research output, likely due to better funding opportunities and established research infrastructure.

### Geographical distribution and collaboration patterns

The analysis of institutional contributions reveals that China and the United States are the primary contributors to AKI and sepsis research. China’s high publication output, however, contrasts with its relatively low rate of international collaboration. Conversely, the United States has demonstrated more frequent international partnerships, which may contribute to the higher citation impact of U.S.-based research. This finding suggests that collaborative research could enhance the visibility and impact of scientific outputs in this field, as demonstrated by countries like Australia, which has a high rate of MCP. European countries, particularly Germany, Italy, and the Netherlands, also play crucial roles in global AKI research networks. Such collaborations can help bridge gaps in clinical knowledge and promote the development of standardized guidelines for AKI management in sepsis [22].

### Institutional and author impact

Among institutions, the Pennsylvania Commonwealth System of Higher Education (PCSHE) and the University of Pittsburgh stand out for their significant contributions, highlighting the leadership of U.S. institutions in AKI research. The network of institutions highlights cross-regional collaborations between institutions from well-established research centers. Notably, Chinese institutions exhibit increasing integration into the global research network, underscoring the expanding contribution of Asian researchers to sepsis-induced AKI research. The influence of these institutions is further underscored by key figures like John A. Kellum and Rinaldo Bellomo, whose research has had a substantial impact on the field. Kellum, Bellomo, and their collaborators have been instrumental in advancing our understanding of sepsis-induced AKI pathophysiology, biomarkers, and treatment approaches. The prominence of these authors reflects the importance of sustained research efforts and collaborations in addressing the complex challenges posed by AKI in septic patients.

### Focus on biomarkers and emerging technologies

Our analysis also identifies a growing interest in biomarkers for early diagnosis and prognosis of AKI, with NGAL and procalcitonin as key indicators. These biomarkers facilitate early detection, which is crucial in sepsis-induced AKI, where timely intervention can significantly improve patient outcomes [9,10,23,24]. Additionally, the recent emergence of machine learning as a keyword suggests an increasing interest in leveraging AI to improve diagnostic accuracy and personalize treatment strategies. Machine learning applications could offer new insights into disease prediction, risk stratification, and outcome prediction in AKI, reflecting a shift toward precision medicine in critical care [25,26].

### Evolution of research themes and methodological advances

Keyword co-occurrence and citation trend analyses reveal a distinct evolution in AKI research themes over the past two decades. Early research primarily focused on epidemiology, mortality risk factors, and renal replacement therapy. However, in recent years, there has been a noticeable shift toward biomarkers, precision medicine, and machine learning applications. The co-occurrence network shows that terms related to ‘renal biomarkers’ and ‘risk stratification’ have gained prominence, reflecting growing interest in early AKI detection. Temporal analysis also highlights the emergence of big data and AI, signaling a methodological shift toward computational modeling for risk prediction. This transition underscores how advancements in data analytics and omics technologies are shaping the future of AKI research.

### Limitations and future directions

Despite these insights, this study has limitations, including its reliance on data from the WoSCC, which may exclude relevant articles from other databases. Additionally, we only included articles and reviews published in English, excluding valuable research available in other languages and formats.

Looking ahead, the trends identified in this analysis suggest that future research in AKI should focus on further elucidating the molecular pathways involved in sepsis-induced AKI and developing targeted therapies to modulate these pathways. The integration of machine learning and other advanced analytical tools could also play a pivotal role in personalizing treatment for AKI patients, particularly in the ICU setting. Collaborative efforts among countries, institutions, and key authors will be essential in driving these advancements and addressing the global burden of AKI in critically ill patients.

## Conclusion

This bibliometric analysis provides important insights into the evolving landscape of AKI research, particularly in the context of sepsis-induced AKI. Collaborative efforts will be essential in addressing knowledge gaps and ensuring consistent, evidence-based care across diverse healthcare settings. Moving forward, biomarker development and the integration of AI-driven technologies should be prioritized to enhance early diagnosis and personalize treatment for AKI patients. Machine learning, in particular, offers tremendous potential for improving diagnostic accuracy, predicting disease progression, and optimizing treatment strategies. Additionally, multinational research partnerships should be fostered to enhance knowledge dissemination and promote the development of universally applicable clinical guidelines for sepsis-induced AKI.

## Supplementary Material

Supplementary table 1.docx
