# Supplementary material for: Global research trends and collaborations in acute kidney injury (AKI) and sepsis: a bibliometric analysis (2004–2024)
Source: Ren Fail. 2025 Apr 24;47(1):2494049. doi: 10.1080/0886022X.2025.2494049 (PMC12035943; doi:10.1080/0886022X.2025.2494049)
Supplement: Supplementary table 1.docx [file IRNF_A_2494049_SM9269.docx]

Supplementary table 1. Institutional Affiliations, and Countries of Authors

| Author | Institution | Country |
| --- | --- | --- |
| Kellum JA | University of Pittsburgh School of Medicine | USA |
| Murugan R | University of Pittsburgh School of Medicine | USA |
| Vaara ST | University of Helsinki and Helsinki University Hospital | Finland |
| Bellomo R | School of Medicine, The University of Melbourne | Australia |
| Bagshaw SM | University of Alberta | Canada |
| Ronco C | San Bortolo Hospital | Italy |
